# Human single-chain variable fragment antibody inhibits macrophage migration inhibitory factor tautomerase activity

**DOI:** 10.3892/ijmm.2014.1622

**Published:** 2014-01-13

**Authors:** MAYURI TARASUK, ORNNUTHCHAR POUNGPAIR, DUANGPORN UNGSUPRAVATE, KUNAN BANGPHOOMI, WANPEN CHAICUMPA, PA-THAI YENCHITSOMANUS

**Affiliations:** 1 Division of Molecular Medicine, Department of Research and Development, Faculty of Medicine Siriraj Hospital, Mahidol University, Bangkok, Thailand; 2 Department of Biochemistry, Faculty of Sciences, Kasetsart University, Bangkok, Thailand; 3 Laboratory for Research and Technology Development, Department of Parasitology, Faculty of Medicine Siriraj Hospital, Mahidol University, Bangkok, Thailand

**Keywords:** macrophage migration inhibitory factor, tautomerase activity, human single-chain variable fragment antibody, inflammation, inflammatory disease

## Abstract

Macrophage migration inhibitory factor (MIF) is a pro-inflammatory cytokine, secreted from a variety of immune cells, that regulates innate and adaptive immune responses. Elevation of MIF levels in plasma correlates with the severity of inflammatory diseases in humans. Inhibition of MIF or its tautomerase activity ameliorates disease severity by reducing inflammatory responses. In this study, the human single-chain variable fragment (HuScFv) antibody specific to MIF was selected from the human antibody phage display library by using purified recombinant full-length human MIF (rMIF) as the target antigen. Monoclonal HuScFv was produced from phage-transformed bacteria and tested for their binding activities to rMIF by indirect enzyme-linked immunosorbent assay as well as to native MIF by western blot analysis and immunofluorescence assay. The HuScFv with highest binding signal to rMIF also inhibited the tautomerase activities of both rMIF and native MIF in human monoblastic leukemia (U937) cells in a dose-dependent manner. Mimotope searching and molecular docking concordantly demonstrated that the HuScFv interacted with Lys32 and Ile64 in the MIF tautomerase active site. To the best of our knowledge, this is the first study to focus on MIF-specific fully-human antibody fragment with a tautomerase-inhibitory effect that has potential to be developed as anti-inflammatory biomolecules for human use.

## Introduction

Macrophage migration inhibitory factor (MIF) was identified in 1966 as the first T-cell cytokine inhibiting random movement of macrophages (1). The biological and physiological activities of human MIF were elucidated from studies on recombinant human MIF generated by molecular techniques. Structural analysis demonstrated that MIF is a homotrimeric molecule with similar characteristics and activities to the human *D*-dopachrome tautomerase (D-DT) enzyme (2). MIF is considered a multifunctional protein constitutively expressed and stored in preformed cytoplasmic pools in several immune cells including monocytes, macrophages, T and B lymphocytes, eosinophils, neutrophils, and dendritic cells and is rapidly released in response to stimuli. In addition, MIF has been identified in various tissues and organs such as lung, the epithelial lining of the skin, gastrointestinal and genitourinary tracts (reviewed in ref. 3).

MIF plays a pivotal role in immune inflammatory responses by acting as a major mediator in the counter-regulation of the immunosuppressive effects of glucocorticoids. As it is an upstream regulator, MIF exerts its effects in innate and adaptive immune systems by the activation of leukocyte recruitment and initiation of the inflammatory cascade for the release of other pro-inflammatory cytokines and mediators such as interleukin (IL)-1β, IL-2, IL-6, IL-8, TNF-α, nitric oxide and prostaglandin E2 (3–5). Furthermore, MIF inhibits p53-mediated apoptosis of immune cells during inflammatory activation (6). MIF biological activity correlates with several catalytic activities such as D-DT (7), phenylpyruvate tautomerase (8), and thiol-protein oxidoreductase (9). However, its physiological substrate has yet to be identified.

Clinical studies have identified the correlation between MIF levels in plasma or affected tissue and disease severity in a number of inflammation-associated diseases. Elevated levels of MIF have been detected in sepsis (10) as well as numerous infectious diseases with exaggerated immune response such as dengue hemorrhagic fever (11,12) and influenza virus infection (13). An increase of secreted MIF has also been observed in a number of inflammatory-mediated autoimmune and also metabolic diseases (14–19). In addition, MIF has a direct relationship with cancer growth and progression (20). Therefore, MIF has become a promising biomarker for diagnosis and an attractive therapeutic target for the treatment of a wide variety of diseases.

Inhibition of MIF activities by small molecules or neutralizing antibodies has become crucial in identifying therapeutic strategies to alleviate immuno-inflammatory disorders in humans. It has been well documented that interference of MIF tautomerase activity readily reduces inflammatory-mediated pathogenesis in various disease models such as sepsis, diabetes and allergic neuritis (21–24). Similar to MIF inhibitors, neutralizing anti-MIF antibodies have been proven to be therapeutically effective in several models of autoimmune and inflammatory diseases (25–29). Recently, antibodies specific to β-sheet structure containing the oxidoreductase motif of MIF have been demonstrated for their protective effects in sepsis model or contact hypersensitivity (30). In the present study, fully human single-chain variable fragment (HuScFv) antibody, which is five-fold smaller than the normal antibody molecule, specific to MIF has been generated. HuScFv selected from a human antibody phage display library, not only recognizes human MIF, but also neutralizes its tautomerase activity. The results of several biological experiments indicate the potential development of MIF-specific HuScFv for therapeutic or diagnostic applications.

## Materials and methods

### Cell culture and native protein preparation

Human monoblastic leukemia (U937) cells were cultured in RPMI-1640 medium (Gibco, Grand Island, NY, USA) supplemented with 10% FBS (vol/vol), 2 mM L-glutamine and 100 U/ml antibiotics at 37°C in a 5% CO_2_ atmosphere. Native MIF in U937 cell lysate was used as an antigen in the western blot analysis and experiments regarding tautomerase activity.

### Production and purification of recombinant human MIF (rMIF)

Full-length human *MIF* was amplified from kidney Matchmaker cDNA library (Clontech, Mountain View, CA, USA) by using *MIF*-specific primers (forward, 5′-CGG GAT CCA TGC CGA TGT TCA TCG TAA ACA CC-3′ and reverse, 5′-CCG CTC GAG GGC GAA GGT GGA GTT GTT C-3′). *Bam*HI and *Xho*I endonuclease restriction sites (underlined) were incorporated for DNA cloning purposes. The amplicon was digested and ligated into pET21a(+) vector (Novagen, Darmstadt, Germany) and introduced into *E. coli* BL21(DE3). A colony of transformant *E. coli* carrying *MIF* was induced by IPTG for rMIF production. Polyhistidine-tagged-rMIF was purified by TALON™ Metal Affinity Resin (Clontech) under native conditions. The purity of rMIF was determined by 15% SDS-PAGE and Coomassie Brilliant Blue G-250 (Sigma, St. Louis, MO, USA) staining.

### Phage bio-panning

Phage clones carrying MIF-specific HuScFv were selected from the human antibody phage display library by bio-panning procedure (31). Purified rMIF (1 μg) was coated into microtiter wells and phage library (100 μl containing ~10^11^ pfu) was added. Phages exhibiting HuScFv that bound to rMIF were rescued by *E. coli* HB2151 infection and selected on selective agar plates (LB containing 100 μg/ml ampicillin and 2% glucose). Individual phagemid-transformed *E. coli* clones were screened for the presence of *huscfv* in a phagemid vector by colony PCR using phagemid-specific primers induced for monoclonal HuScFv production, as previously described (31). Bacterial lysates were detected for E-tagged HuScFv by western blot analysis using anti-E-tag polyclonal antibody (Abcam, Cambridge, UK) followed by HRP-conjugated swine anti-rabbit Ig (Dako, Glostrup, Denmark) and DAB substrate.

### Screening of MIF-specific HuScFv by indirect enzyme-linked immunosorbent assay (ELISA)

Indirect ELISA was performed to determine the binding of monoclonal HuScFv to rMIF. The wells of ELISA plate were coated with 1 μg purified rMIF or BSA (negative antigen control) at 37°C overnight. After washing and blocking the wells, HuScFv-containing preparations (1 mg in 100 μl) were added individually to both rMIF and BSA wells and incubated at 37°C for 2 h. HuScFv binding to rMIF was detected by rabbit anti-E-tag polyclonal antibody followed by HRP-conjugated swine anti-rabbit IgG. Enzymatic reaction was developed following the addition of TMB substrate (Invitrogen, Camarillo, CA, USA) and 1 N HCl. Color of the content in the wells was measured at OD_450nm_ using ELISA reader (Multiskan *EX*; Thermo Scientific, Waltham, MA, USA).

### Preparation of purified 6xHis-tagged HuScFv

The Huscfv sequence in the phagemid vector of the selected *E. coli* clone was subcloned into modified pET23b(+) vector and introduced into *E. coli* BL21(DE3) by transformation (32). Bacterial transformants containing pET23b(+)-*huscfv* were induced with IPTG for the production of monoclonal 6xHis-tagged HuScFv. The HuScFv in the bacterial lysate was purified using TALON Metal Affinity Resin and prepared in 1X PBS (pH 7.4) by dropwise dialysis prior to use.

### Determination of the binding activity of HuScFv to native MIF

Western blot analysis and immunofluorescence assay were performed to determine the binding activity of HuScFv to native MIF in human U937 cells. U937 whole cell lysate (40 μg) was separated on SDS-PAGE and transferred onto nitrocellulose membrane. Polyhistidine-tagged HuScFv was added to the membrane and subsequently detected by mouse anti-His antibody. The reactive band of HuScFv-MIF immune complexes was revealed by adding AP-conjugated goat anti-mouse Ig and BCIP/NBT colorimetric substrate, respectively. The irrelevant HuScFv (dengue virus capsid protein-specific HuScFv) and mouse anti-MIF polyclonal antibody were used as negative and positive antibody controls, respectively.

Immunofluorescence assay was used to demonstrate and localize the interaction of HuScFv to cellular MIF in U937 cells. The cells were fixed with 4% paraformaldehyde and permeabilized with 0.2% Triton X-100. After blocking, the cells were incubated with purified HuScFv (1 μM) at 37°C for 2 h in a humidified chamber. The HuScFv-MIF interaction was revealed by adding a mixture of mouse anti-His antibody and rabbit anti-MIF polyclonal antibody. The cells were then incubated with a mixture of Alexa Flour 488-conjugated goat anti-mouse Ig (Molecular Probes, Carlsbad, CA, USA), Cy™3-conjugated AffiniPure donkey anti-rabbit Ig (Jackson ImmunoResearch Laboratories, West Grove, PA, USA), and anti-nuclear staining reagent (Hoechst; Molecular Probes) to localize HuScFv, endogenous MIF, and nuclear DNA, respectively. Fluorescence images were visualized by using a laser scanning confocal microscope (LSM 510 META; Carl Zeiss, Jena, Germany).

### Neutralization of MIF tautomerase activity by HuScFv

Recombinant MIF and native MIF in U937 cell lysates were analyzed for its inherent tautomerase activity as previously described with slight modifications (33). The enzymatic reaction was initiated at 25°C by adding 20 μl of the dopachrome methyl ester substrate (2 mM L-3,4-dihydroxyphenylalanine methyl ester and 4 mM sodium periodate) to a cuvette containing 200 μl of either rMIF (30 μM) or native MIF in U937 cell lysate (6 μg) prepared in tautomerase assay buffer (50 mM potassium phosphate, 1 mM EDTA, pH 6.0). The activity was determined by the semi-continuous reduction of OD_475nm_ at 60-sec interval for 300 sec using a spectrophotometer (Shimadzu, Kyoto, Japan). The neutralization of tautomerase activity by MIF-specific HuScFv was determined by pre-incubation of HuScFv with rMIF or native MIF in U937 cell lysate at 37°C for 1 h prior to the enzymatic reaction was initiated. Irrelevant HuScFv was used as a negative antibody control. Heat-denatured rMIF and U937 cell lysate were used as tautomerase activity-negative controls. Results were presented as the mean values from three independent experiments.

### HuScFv mimotope searching and in silico analysis of HuScFv-MIF interaction

Mimotope of MIF-specific HuScFv was determined by using Ph.D.-12™ Phage Display Peptide Library (New England Biolabs; Ipswich, MA, USA) as previously described (34). Purified HuScFv was used as the target for selection of phage clones displaying 12-mer peptide (mimotope). After three rounds of bio-panning, genomic DNA from a number of phage clones displaying peptides bound to MIF-specific HuScFv was sequenced and the 12-mer peptide sequences were deduced. Consensus mimotope was obtained by multiple alignments of the deduced peptide sequences. The mimotope was aligned to the MIF protein sequence and residues on the MIF molecule matching with the obtained mimotope were considered as the HuScFv epitope.

Molecular modeling of the antibody was initiated via BLAST search analysis. The highest identity sequence with 3D structure was used as a template for homology modeling by using the modeler module embedded in the Discovery Studio 2.5 program (Accelrys Software Inc., San Diego, CA, USA). The three-dimensional structure of MIF was obtained from the PDB database (PDB entry 1GD0) and the crystal structure of a human anti-SARS spike protein antibody, 80R (PDB entry 2GHW) was used as a template for modeling of the HuScFv molecule. The constructed models were accessed for the quality of structure via the Ramachandran plot. The plot was generated using PROCHECK v3.4 program (University College London, London, UK) (35). Subsequently, the docked poses were subjected to structural refinements by using RDOCK module embedded in Discovery Studio 2.5 program.

### Statistical analysis

Data were analyzed using GraphPad Prism Software 4.0 (GraphPad Software, Inc., San Diego, CA, USA) and presented as the mean ± SEM. Significant differences were determined by one-way ANOVA (P<0.05) and Tukey’s HSD test.

## Results

### Production of rMIF with inherent enzymatic activity

A full-length cDNA encoding MIF was amplified from human cDNA library (345 bp). Following DNA sequencing, the sequence was deposited into the GenBank database (accession no. JQ846015). It showed 100% homology to the human MIF-coding sequence previously reported in the database (NM_002415.1). Recombinant MIF was abundantly expressed after induction of the bacterial clone carrying recombinant plasmid. Polyhistidine-tagged rMIF was purified as a soluble protein at 12.5 kDa. Tautomerase enzymatic activity, a distinct pathogenesis-related property of MIF, was verified as the reduction of dopachrome methyl ester substrate at OD_475nm_ (33). Purified rMIF exhibited *in vitro* tautomerase activity, suggesting that rMIF presented inherent physiological function, and retained enzymatic epitope similar to the native molecule (data not shown).

### Selection and screening of MIF-specific HuScFv

**Figure 1:**
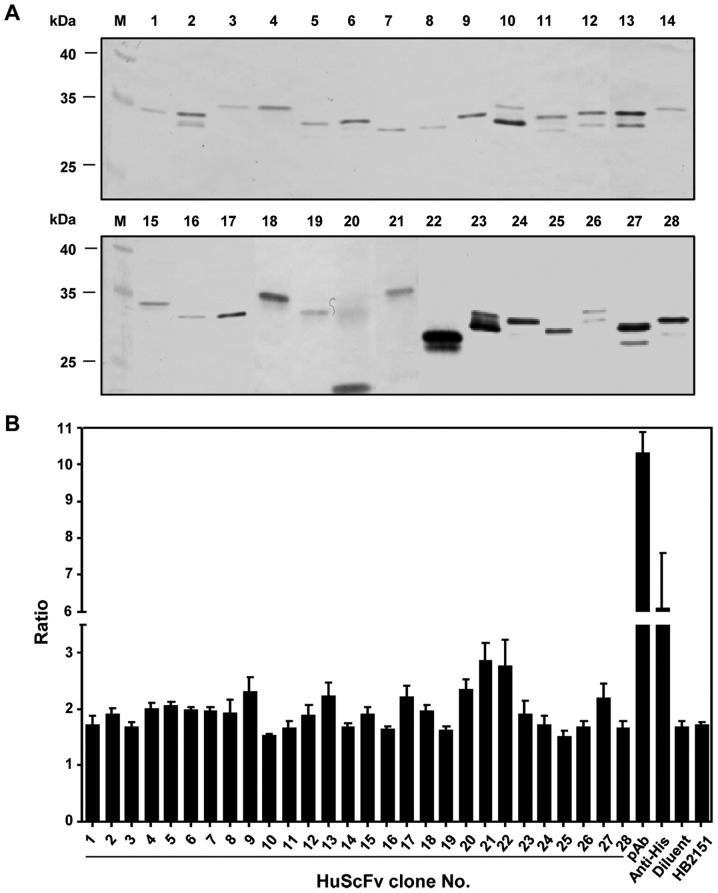
Screening of phage clones displaying migration inhibitory factor (MIF)-specific human single-chain variable fragment (HuScFv) and determination of HuScFv binding activity to recombinant human MIF (rMIF). (A) After bio-panning, monoclonal soluble HuScFv produced from *huscfv* phagemid-transformed HB2151 *E. coli* was detected by western blot analysis using the anti-E-tag antibody. The result revealed HuScFv (20–35 kDa) production from 28 *E. coli* clones. (B) Indirect enzyme-linked immunosorbent assay (ELISA) was used to determine the binding activities of 28 HuScFv clones to rMIF. The results were presented as an average ratio of the binding signal of HuScFv-rMIF compared to HuScFv-BSA (OD_450nm_ of HuScFv-rMIF/OD_450nm_ of HuScFv-BSA) ± SEM. Mouse anti-MIF polyclonal antibody and anti-His antibody were used as positive antibody controls. Non-HuScFv-producing *E. coli* lysate served as the negative antibody control.

**Figure 2:**
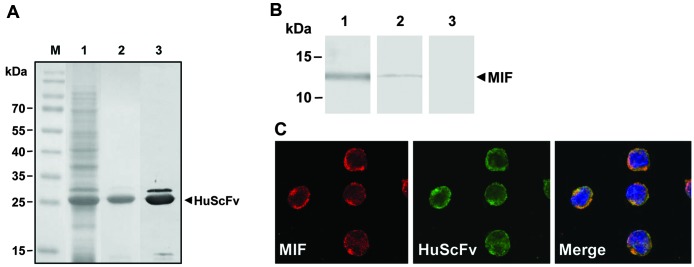
Production of purified migration inhibitory factor (MIF)-specific human single-chain variable fragment (HuScFv) and its binding activity against native MIF. (A) SDS-PAGE and Coomassie Brilliant Blue G-250 staining patterns of *E. coli* lysate containing 6xHis-tagged HuScFv of clone no. 22 (lane 1) and the affinity-purified HuScFv (lane 2) were revealed. Western blot analysis indicated HuScFv of clone no. 22 (25 kDa) as detected with anti-His antibody (lane 3). (B) Western blot analysis for determining the binding activity of HuScFv to native MIF. U937 lysate (40 μg) was blotted onto nitrocellulose membrane and detected with either i) mouse anti-MIF polyclonal antibody (lane 1), ii) MIF-specific HuScFv followed by mouse anti-His antibody (lane 2), or iii) dengue capsid-specific HuScFv followed by mouse anti-His antibody (lane 3). Reactive bands of native MIF were observed at size 12.5 kDa. (C) HuScFv co-localized with native MIF in U937 cells. Cells were fixed and immunostained with MIF-specific HuScFv as observed in green fluorescence. Rabbit anti-MIF polyclonal antibody indicated intracellular MIF (red fluorescence). The combined image demonstrated co-localization of MIF-specific HuScFv and native MIF as yellow fluorescence throughout the cytoplasm of U937 cells.

Purified rMIF was used as the antigen for selection of phages carrying MIF-specific HuScFv by bio-panning of the human antibody phage display library. A number of *E. coli* clones carrying *huscfv* were obtained and randomly tested for the production of monoclonal soluble HuScFv. Among *huscfv*-positive transformants, 28 clones (35%) produced HuScFv (20–35 kDa) as detected by western blot analysis using anti-E-tag antibody (Fig. 1A). These clones were individually screened for their binding activities to rMIF by indirect ELISA. Seventeen clones exhibiting ELISA signal ratios (OD_450nm_ of HuScFv-rMIF binding/OD_450nm_ of HuScFv-BSA binding) were higher than the ELISA signal ratio identified in the negative control (Fig. 1B). *Huscfv* of *E. coli* clones exhibiting high binding activity were subcloned into a modified pET23b(+) vector for benefits in HuScFv purification and detection in subsequent experiments. Only the HuScFv of clone no. 22 (~25 kDa) was produced in an sufficient amount and appropriate purity for subsequent experiments as revealed by SDS-PAGE and western blot analysis detected with anti-6xHis antibody (Fig. 2A).

### Characterization of MIF-specific HuScFv

The HuScFv no. 22 was demonstrated for its binding activity to native MIF by western blot analysis and immunofluorescence staining. Western blot analysis revealed a reactive band of native MIF from U937 cell lysate bound to HuScFv at the same size as that of the native human MIF protein (12.5 kDa), similar to the positive antibody control (mouse anti-MIF pAb), while the irrelevant HuScFv showed no reactive band (Fig. 2B). Immunofluorescence staining confirmed the binding activity of HuScFv to intracellular MIF in U937 cells. Co-localization of MIF and HuScFv throughout the cytoplasm of U937 cells was clearly observed under confocal microscopy (Fig. 2C).

### Inhibition of MIF tautomerase activity by HuScFv

**Figure 3:**
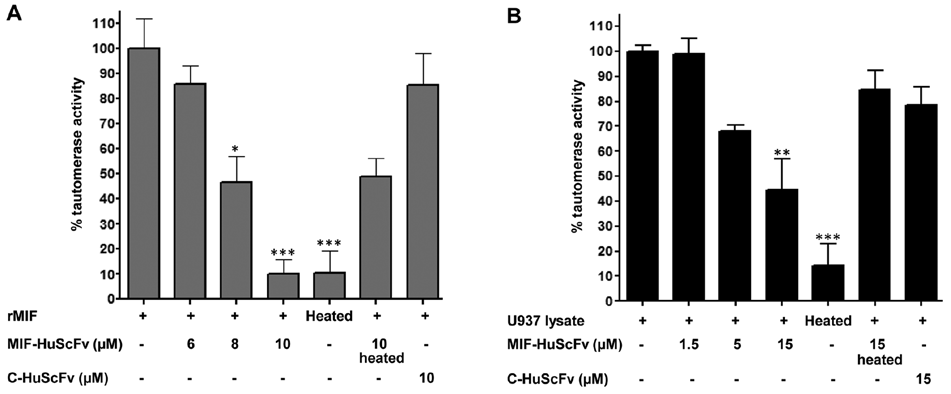
Neutralization of migration inhibitory factor (MIF) tautomerase activity by human single-chain variable fragment (HuScFv). Relative tautomerase activity of (A) recombinant human MIF (rMIF) and (B) native MIF from U937 cells in the presence of HuScFv at various concentrations were shown. Heat-denatured MIF, heat-denatured HuScFv, and irrelevant HuScFv (C-HuScFv; dengue virus capsid-specific HuScFv) were used as negative controls. Data are presented as mean ± SEM of three independent experiments (^*^P<0.05, ^**^P<0.01 and ^***^P<0.001 vs. control; rMIF or U937 cell lysate alone).

Inhibitory effect of HuScFv on MIF dopachrome tautomerase activity was determined. Following pre-incubation with HuScFv, tautomerization reaction indicated by substrate decolorization of both rMIF and native MIF was reduced compared to that of the non-HuScFv control reaction. Increased inhibition was evident with the increasing amount of HuScFv present in the reaction. At the end-point of rMIF-mediated enzymatic reaction, HuScFv levels at concentrations of 6, 8 and 10 μM markedly reduced tautomerase activity to 86, 46 and 10%, respectively, compared to the non-HuScFv control reaction (Fig. 3A). On the other hand, heat-denatured and irrelevant HuScFv did not significantly affect the enzymatic activity of rMIF.

The HuScFv selected from rMIF was also tested for its inhibition of tautomerase activity mediated by native MIF in human monoblastic leukemia (U937) cells. Tautomerase activity of MIF in U937 cells was reduced as MIF-specific HuScFv was added prior to initiation of enzymatic reaction. As the HuScFv concentration increased (1.5, 5 and 15 μM) the average percentages of tautomerase activity were reduced to 99, 68 and 44%, respectively (Fig. 3B). By contrast, denatured MIF-specific and irrelevant HuScFv failed to hinder tautomerase activity.

### Mimotope searching and in silico analysis of HuScFv-MIF interaction

Human MIF-HuScFv interaction was analyzed by means of mimotope searching and molecular docking. In mimotope searching, 20 phage clones carrying peptide mimotopes of HuScFv were obtained and the 12-mer consensus mimotope sequence was ‘MSTPLGQYTGTK’. HuScFv mimotope was aligned on the MIF sequence and it was found to locate at the residues 22–33 of the human MIF-residing tautomerase active site.

**Figure 4:**
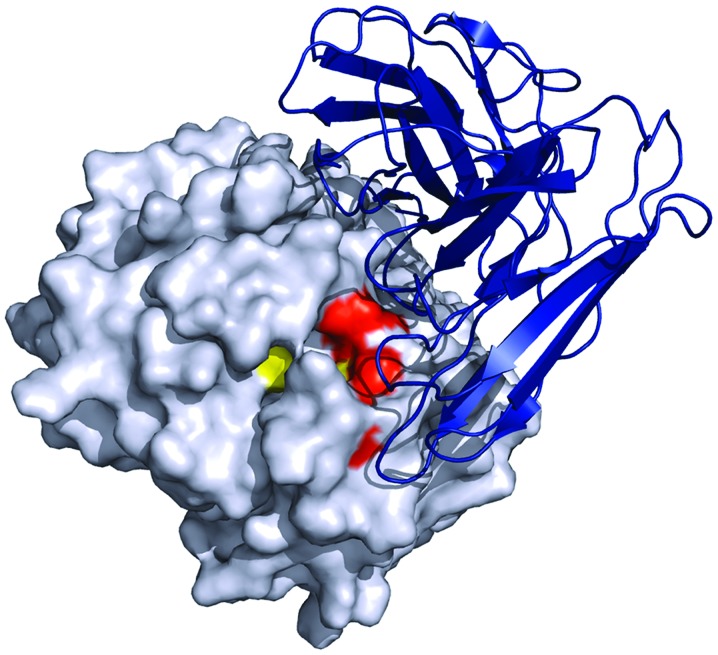
3D structure of migration inhibitory factor (MIF)-human single-chain variable fragment (HuScFv) complexes. Complex structure of HuScFv (blue ribbon) and trimeric MIF (gray surface) is shown. The tautomerase active site residues are shown in yellow and red surfaces. Mimotope searching and molecular docking results indicated that HuScFv interacted with Lys32 and Ile64 within the tautomerase active site (yellow surface) in the MIF molecule.

In molecular docking, HuScFv was modeled using a human anti-SARS spike protein antibody with 75% sequence similarity to the template. Ramachandran plot of the modeled HuScFv revealed the acceptable disallowed region (1.0%), indicating that the modeled structure is appropriate for the molecular docking analysis. The docked pose calculated by ZDOCK and RDOCK shown in Fig. 4, exhibited low-binding energy of HuScFv-MIF binding (−17.493 kcal/mol) at residues Lys32 and Ile64 of the MIF tautomerase active site.

## Discussion

The correlation of increased MIF levels in plasma with severity of inflammatory diseases has been previously reported. Thus, MIF is considered a promising biomarker (36) and therapeutic target for these diseases. It has been shown that interference or abrogation of enzymatic activity of MIF rescued individuals that succumbed to inflammation-mediated pathogenesis (21,37). A number of inhibitors and neutralizing antibodies have been introduced for the inhibition of MIF activity. Recently, Kerschbaumer *et al* (30) generated fully human anti-MIF antibodies by selection from a phage display library and extensively analyzed these antibody molecules *in vitro* and *in vivo*. Results of that study showed that only the antibody-binding epitopes within amino acids 50–68 or 86–102 of the MIF molecule, the regions forming a β-sheet structure in the MIF oxidoreductase motif, exerted protective effects in models of sepsis or contact hypersensitivity. Findings of this study have demonstrated that fully human antibody fragments specific to MIF inhibited the enzymatic activity of MIF and presented therapeutic potentials. Identification of HuScFv specific to MIF is also crucial. Unlike the previous study (30), we identified and reported HuScFv that inhibits tautomerase activity of MIF.

In this study, we initially amplified the coding sequence of human MIF from the human cDNA library, which showed 100% homology to the human MIF-coding sequence previously reported in the NCBI database (NM_002415.1). The rMIF generated from the human MIF cDNA construct exhibited tautomerase activity, suggesting that it carried a native structure and active enzymatic sites; thus, it was suitable for additional use as the target antigen for selection of MIF-specific HuScFv from the human antibody phage display library. This library, constructed in our laboratory, has previously been used for the selection of a number of neutralizing HuScFv specific to different pathogenic antigens (31,32,38,39). However, it has not been previously used for the selection of HuScFv specific to a human protein.

Naturally, immature B lymphocytes carrying antibodies specific to human autologous proteins are eliminated during B-lymphocyte maturation; consequently, they are absent from the pool of circulating lymphocytes. However, results of this study have demonstrated that the human antibody phage display library prepared from human B lymphocytes is composed of HuScFv specific to human MIF that should be deleted from the matured B lymphocytes. This finding is attributable to the fact that in the generation step of human antibody phage display library the heavy and light chains (*VH* and *VL*) of antibody genes were individually amplified and allowed to randomly fuse to form *HuScFv*. Thus, the combination of VH and VL that did not exist in nature was available in the *in vitro* preparation of synthetic antibody phage display library. This explains the fact that a smaller number of *huscfv*-positive phagemid-transformed *E. coli* from MIF-bio-panning (29%) was obtained when they were compared to those from the selection of HuScFv specific to pathogenic proteins reported in previous studies (59–100%) (31,34,38).

Some of the HuScFv clones obtained exhibited strong binding activity to rMIF as determined by indirect ELISA. The HuScFv with high binding signals were produced and purified. Only HuScFv of clone no. 22 yielded sufficient amount with appropriate purity, and it was subsequently characterized by results of different experiments. Western blot analysis revealed that HuScFv no. 22 bound to native MIF protein prepared from the monocytic cell line, U937. Moreover, immunofluorescence staining revealed co-localization of HuScFv and native MIF in the cytoplasm of U937 cells. These results readily indicate that although it was selected by rMIF, the HuScFv molecules interacted with its target MIF antigen in the native form.

The tautomerase-active site is essential for the pro-inflammatory activity of MIF; thus, attempts have been made to develop tautomerase-neutralizing inhibitors (21,40). A number of these inhibitors have also been proven beneficial for therapeutic efficacy in MIF-associated diseases (24,40–42). In the present study, inhibition of dopachrome tautomerization was performed to assess inhibition of MIF tautomerase activity mediated by HuScFv. Subsequent to pre-incubation with MIF-specific HuScFv, the tautomerization activity of rMIF and native MIF, as detected by substrate decolorization, was reduced in a dose-dependent manner, when compared to that of the non-HuScFv control reaction, indicating its specific inhibition to MIF enzymatic activity. The finding that HuScFv inhibited the tautomerase activity of rMIF to a greater extent than that of native MIF may indicate the presence of other proteins containing the tautomerase activities in U937 cell lysate (2). *In vitro* neutralization of MIF-mediated enzymatic activity suggested the possibility of using HuScFv to reduce MIF-induced pro-inflammatory reactions, similar to the inhibitors or specific monoclonal antibodies as demonstrated in previous studies (21,43). Experiments on *in vivo* neutralization tests are required to evaluate anti-inflammatory activities of MIF-specific HuScFv (30).

The HuScFv and MIF interaction was elaborated by the target epitope searching and molecular docking approaches. The 12-mer consensus mimotope sequence of MIF-specific HuScFv is located at the amino acid residues 22–33 of human MIF residing within the tautomerase active site. Molecular docking also revealed that the interaction between HuScFv and MIF spontaneously occurred with low-binding energy at residues Lys32 and Ile64 of the MIF tautomerase active site. This finding strongly supports the previous description of tautomerase catalytic residues including Pro1, Lys32, Ile64, Tyr95 and Asn97 (40). The phenomenon of the HuScFv-mediated inhibition of MIF tautomerase activity is similar to that exerted by the ISO1 chemical inhibitor (40).

In conclusion, HuScFv selected by using recombinant human MIF readily recognized native MIF present in human monoblastic leukemia (U937) cells. The antibody also neutralized tautomerase activity mediated by both rMIF and native MIF as it interacted with catalytic residues residing in the tautomerase active site of the MIF molecule. The inhibitory effect was elaborated by mimotope searching and the molecular docking approaches. MIF-specific HuScFv, with its fully-human format and small size, should be investigated in subsequent experiments to demonstrate its anti-inflammatory activity and its ability to develop into a therapeutic molecule for the treatment of human inflammatory diseases.
